# Comparison of Egg Yolk and Soybean Phospholipids on Hepatic Fatty Acid Profile and Liver Protection in Rats Fed a High-Fructose Diet

**DOI:** 10.3390/foods10071569

**Published:** 2021-07-06

**Authors:** Mingyu Yin, Ryosuke Matsuoka, Yinci Xi, Xichang Wang

**Affiliations:** 1 College of Food Science and Technology, Shanghai Ocean University, No. 999, Huchenghuan Rd, Nanhui New City, Pudong New District, Shanghai 201306, China; yin214300841@163.com (M.Y.); ryosuke_matsuoka@kewpie.co.jp (R.M.); ycxi@shou.edu.cn (Y.X.); 2 Shanghai Engineering Research Center of Aquatic-Product Processing and Preservation, Shanghai 201306, China

**Keywords:** hyperlipidemia, phospholipids, fatty acids, lipid metabolism

## Abstract

Perturbed lipid metabolism leads to ectopic lipid accumulation in tissues, such as the liver, thereby causing nonalcoholic fatty liver disease (NAFLD) and negatively influencing circulating lipid profile-inducing dyslipidemia. Phospholipids (PLs) with special biological activity are used to treat chronic diseases such as cardiovascular and cerebrovascular disease. PLs derived from egg yolk and soya bean have significant antioxidant and lipid-lowering abilities. This study examined the therapeutic effects of them on hyperlipidemia using a high-fructose-fed rat model; lipid metabolism and anti-inflammatory effects were also analyzed. The results showed that both egg yolk and soya bean phospholipids (EPLs and SPLs) reduced liver weight, hepatic TG, and MDA content as well as serum ALT, AST, TBA, and CRP levels (*p* < 0.05). The PLs also showed hypolipidemic and anti-inflammatory effects. EPLs and SPLs could inhibit the accumulation of hepatic fatty acids C18:1N9C, C18:0, and C22:6NS of rats fed a high-fat-and-sucrose diet. The intake of EPLs could significantly increase acetylcholine content in the blood and brain tissue. Histological examination showed that PLs intake could ameliorate the damage to liver tissue. This study suggested that EPLs and SPLs had a certain capacity of hypolipidemic and liver protection, and the therapeutic benefits of EPLs tended to be more effective than that of soybean phospholipids.

## 1. Introduction

Chronic diseases such as coronary heart disease, diabetes, nonalcoholic fatty liver disease (NAFLD), and cancer seriously threaten human health and reduce family happiness indices [1,2]. Perturbed lipid metabolism leads to ectopic lipid accumulation in tissues, such as the liver, thereby causing NAFLD and negatively influencing circulating lipid profile-inducing dyslipidemia [3]. As problems with obesity become more serious, the risks of being afflicted with chronic diseases increase.

Hyperlipidemia represents several different disorders of lipid metabolism related to increased production or delayed degradation of atherogenic lipoprotein particles, or decreased synthesis or increased degradation of protective lipoprotein particles [4]. These alterations lead to pathological changes in the lipid levels in various tissues of the body. The liver is essential for fat catabolism [5]. Several studies have shown that the fat composition of the liver in hyperlipidemia patients is significantly different from that of a normal liver [6,7]. The activity of enzymes in hyperlipidemia patients related to liver health status (e.g., alanine aminotransferase (ALT) and aspartate aminotransferase (AST)) also varies greatly compared with healthy populations. Additionally, a number of studies indicated that marine fish oils, peptides, and polyphenols in natural substances can help restore serum lipid status, decrease the activity of transaminase, and reduce the occurrence of chronic disease [8].

Phospholipids (PLs) are versatile biological macromolecular substances. Cardiovascular and cerebrovascular diseases, including Alzheimer’s disease, could benefit from PLs [9]. The mechanism of action of PLs is via enhancement of excretion of cholesterol in bile and regulation of cholesterol ester transfer protein (CETP) and lipoprotein metabolism [10,11]. PLs and cholesterol are catalyzed by cholesterol ester acyltransferase (LCAT) to form cholesterol esters and lysophospholipids, which further promotes cholesterol metabolism [4]. Egg yolk PLs (EPLs) and soybean PLs (SPLs) are common phospholipids currently on the market. They are typical representatives of animal- and plant-derived PLs. Both sources are hypoglycemic and lipid-lowering [12]. Further, both reduce the risks of cancer, lessen oxidative stress, and enhance immunity [13]. However, to the best of our knowledge, there have been few comparative investigations on the impact of EPLs and SPLs on lipid metabolites and changes in liver fatty acid profiles caused by a high-fructose diet (HFD).

The aim of the present research was to explore whether 2.0% EPLs or SPLs fed to Sprague Dawley (SD) rats with an HFD to develop lipid metabolic dysfunction might differently affect the blood lipid status and liver fatty acid profile. We further aimed to identify the therapeutic benefits of these sources of PLs on hyperlipidemia.

## 2. Experimental

### 2.1. Materials

EPLs were obtained from the Kewpie Corporation (Tokyo, Japan); SPLs were purchased from Tokyo Chemical Industry Co., Ltd. (Tokyo, Japan). N_2_ was obtained from Shanghai Li Dan Industrial Gases Ltd. (Shanghai, China). Organic solvents C19:0 and C19:0, fatty acid methyl esters (FAMEs), and the mixture of 37 FAMEs were purchased from Shanghai ANPEL Scientific Instrument Co., Ltd. (Shanghai, China). According to the manufacturer, all FAMEs in the mixture were in equal amounts based on equal weight (i.e., 2.63% of the mixture). Oil Red O and hematoxylin and eosin (H&E) were obtained from Shanghai Lanji Technological Development Co. Ltd. (Shanghai, China).

### 2.2. Animals and Diets

Three -week-old male Sprague Dawley rats (45.0–55.0 g) were obtained from Shanghai Jiesijie Experimental Animal Co., Ltd. (Shanghai, China). Rats were individually housed in an air-conditioned room with a 12 h light/dark cycle, a constant temperature of 23 ± 2 °C, and relative humidity of 65% ± 15%. Rats were given free access to water and diet. The protocols were approved by the ethical committee of experimental animal care at Shanghai Ocean University (Shanghai, China).

Rats were divided into four groups and fed the following four diets (Table 1): commercial standard chow (control group), an HFD (model group), an HFD containing 2% egg PLs (egg yolk phospholipids group, EP), and an HFD containing 2% soy PLs (soybean phospholipids group, SP), and each group contained 6 animals. The model group diet was prepared using the AIN-76 formula. The TC content of the model, EP, and SP diets was approximately 0.1%. After 4 weeks of feeding, rats were sacrificed after a 12 h overnight fasting. Blood was collected and serum was separated. Livers were quickly excised and frozen in liquid nitrogen and stored at −80 °C until analysis. Both the handling and killing of all the animals were carried out in accordance with the Guidelines of Experimental Animal Administration published by the State Committee of Science and Technology of the People’s Republic of China and EU Directive 2010/63/EU for animal experiments. Composition and mass percentage of experimental diets are in Table 1.

### 2.3. Body Weight, Food Intake, and Liver, Abdominal Fat, and Brain Weight

At the end of feeding, body weight, food intake, food efficiency (FI), liver weight, and abdominal fat weight were recorded, and FI were calculated as follows: FI was calculated by dividing the total weight gain during the experiment by the total food intake.

### 2.4. Histopathological Observation of the Liver

A portion of the liver was fixed in 4% paraformaldehyde solution. The fixed liver was embedded in paraffin, cut into slices (6 µm thick), and stained with H&E. For Oil Red O staining, a frozen liver sample was processed using a cryostat (CM1950, Leica, Munich, Germany) and stained. The stained areas were observed under an Olympus light microscope and photographed. The samples were fixed at room temperature with 4% formaldehyde (formaldehyde:water, V:V) for 40 h, followed by 20%, 30%, 50%, 70%, 75%, 85%, 95%, and 100% ethanol solution gradient dehydration (the dehydration time at each concentration was 18 min); samples were then freeze-dried for 16 h and vacuum gold-plated. The SEM observation was carried out.

### 2.5. Analysis of Lipid Parameters

We determined serum TC (Beijing Solibao Technology Co. Ltd., Beijing, China), TG (triglyceride E-test Wako; Wako Pure Chemical Industries, Ltd., Tokyo, Japan), and free fatty acid (FFA, Beijing Solibao Technology Co., Ltd., Beijing, China) content, as well as activity of aspartate transaminase (AST, also known as GOT) (Changchun Huili Biotechnology Co., Ltd., Changchun, China) and activity of alanine transaminase (ALT, also known as GPT) (Changchun Huili Biotechnology Co., Ltd., Changchun, China). We also determined high-density lipoprotein and low-density lipoprotein (HDL-C and LDL-C, Changchun Huili Biotechnology Co., Ltd., Changchun, China) levels and glutathione peroxidase activity (GSH-PX, Nanjing Jiancheng Bioengineering Institute, Nanjing, China). Hepatic lipids were extracted with chloroform/methanol (2:1, *v*/*v*) by using the Folch method (Folch, Lee, & Sloane-Stanley, 1957). Hepatic TC and TG were analyzed as described in serum.

### 2.6. Determination of MDA, TBA, CRP Concentration, and SOD Activity

We determined serum TBA (TBARS Assay kit, Cayman Chemical Company, Ann Arbor, MI, USA), CRP (CRP ELISA kit, rat, Immunity Consultants laboratory Inc., Portland, OR, USA), hepatic MDA (Beijing Solibao Technology Co., Ltd., Beijing, China), and SOD activity (Beijing Solibao Technology Co., Ltd., Beijing, China) levels.

### 2.7. Phospholipids and Related Metabolites

PL content in serum, liver, and brain (phospholipid C-test Wako; Wako Pure Chemical Industries, Ltd., Japan) as well as choline/acetylcholine contents in serum, liver, and brain (Quantification kit, BioVision, Milpitas, CA, USA) were determined.

### 2.8. Analysis of Fatty Acids of PLs and Liver

The fatty acid composition in the PLs was measured by the method as previously described in [13]. Briefly, a sample (0.50 g) was processed in 10 mL chloroform/methanol (2:1, *v*/*v*) and 1 mL C19:0/chloroform (1 mg/mL) according to the extraction method [14]. Samples were homogenized and soaked at 4 °C for 24 h. Homogenates were filtered, and 5 mL of 9% sodium chloride solution was added to the filtrate and allowed to stand for 3 h at 4 °C. The supernatant was collected, and the operation was repeated. After obtaining the final supernatant, anhydrous Na_2_SO_4_ was used to remove water. Fat extract was concentrated by rotation in a water bath at 35 °C to a constant weight. In all, 5 mL methanolic NaOH (0.5 mol/mL) was added to the fat extract. The mixture was placed in condensing and concentrating equipment (HWS24, HongLang, Henan, China) and heated at 100 °C for 10 min. Subsequently, 3 mL boron trifluoride–methanol (14% in methanol) was added at 100 °C and stirred for 3 min, followed by the addition of 2 mL n-hexane and was maintained at 100 °C for 2 min. Finally, 10 mL saturated NaCl solution was added to the mixture. Samples were cooled to room temperature (24 °C); the upper n-hexane layer was collected using a 2 mL disposable syringe, purified with a nylon syringe filter (13 mm × 0.22 µm), and stored in a 2 mL thread screw neck vial with a septum (32 mm × 11.6 mm, ANPEL Inc., Shanghai, China) for further analysis.

The sample was then detected by the gas chromatograph (TRACE GC ULTRA, Thermo Fisher Inc., Waltham, MA, USA) equipped with an Agilent (Santa Clara, CA, USA) SP-2560 capillary column (100 m length × 250 µm internal diameter, 0.2 µm of film) and a flame ionization detector (Thermo Fisher Inc., MA, USA) The contents of different fatty acids were determined using the area ratio of the GC peak between internal standard C19:0 and fatty acids from samples.

### 2.9. Statistical Analysis

The results are presented as mean ± SE. All data were subjected to analysis of variance using the SPSS software (version 19.0; SPSS Inc., Chicago, IL, USA). Differences between four groups were tested by one-way ANOVA, followed by Duncan’s test. The level of significance chosen was *p* < 0.05. The figures were drawn using the software Prism 6.0 (GraphPad, San Diego, CA, USA). Multi Experiment Viewer (MeV4.9) software was used for the nonparametric test. Principal component analysis (PCA) and orthogonal partial least-squares discriminant analysis (OPLS-DA) were carried out using the SIMCA-P 14.1 software (Umea, Sweden). Further, all fatty acids were used in volcano plots considering fold change (FC > 1.5) and the statistical *p*-value (*p* < 0.05).

## 3. Results

### 3.1. Fatty Acid Composition

Table S1 shows that the main fatty acid composition of EPLs and SPLs were similar, but the animal origin EPL had a special arachidonic acid (C20:4, ARA). The contents of C16:0, C18:0, and C18:1N9C in EPLs were significantly higher than those in SPLs (*p* < 0.05). The content of linoleic acid (C18:2N6C, LA) in SPLs was significantly higher than that in EPLs (*p* < 0.05).

### 3.2. Effects of Dietary PLs on Body and Tissue Weights

The initial body weights of rats at 4 weeks of age were measured and no significant differences were observed (Table 2). After four weeks of feeding, we evaluated the effects of PLs on growth paraments and tissue weight in rats. The body weight and the food intake markedly increased in HFD rats compared with the control group. The body weight in EP group rats was significantly decreased compared with the control group and SP group (*p* < 0.05). Treatment with PLs effectively attenuated the HFD-induced alterations in body weight, food efficiency, liver, and abdominal fat weight to some different degree. EPLs significantly blocked the significant increase in the body weight, food efficiency, liver weight, and abdominal fat weight compared to the model group, whereas administration of SPLs did not exhibit any alterations in body weight and abdominal fat weight without significant differences compared with the model group, which were significantly higher than the EP group (*p* < 0.05).

### 3.3. Hepatic Histopathological Observations

**Figure 1:**
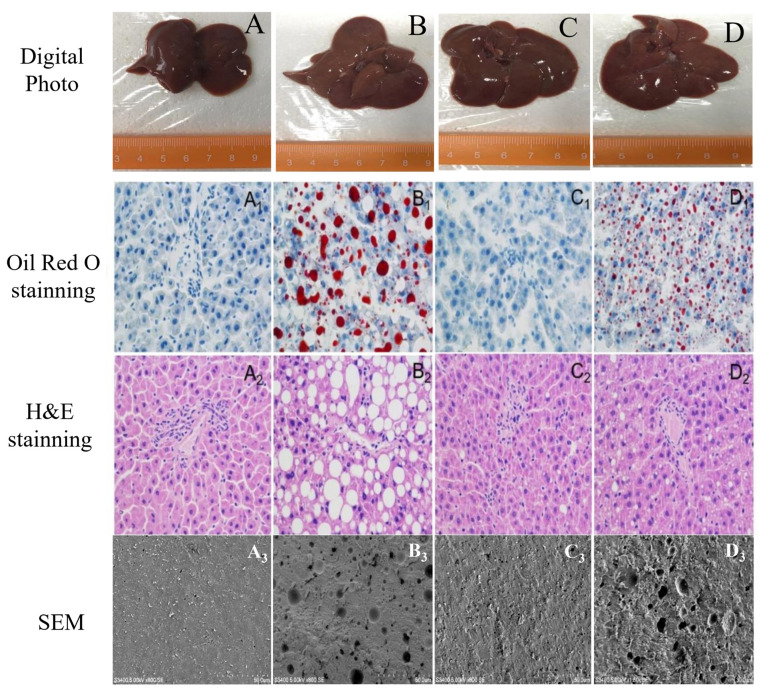
Histopathological alterations in the abdominal fat pad and liver of rats. (**A**): Control group; (**B**): model group; (**C**): EP group; (**D**): SP group. An HFD containing 2% egg PLs (egg yolk phospholipids group, EP), and an HFD containing 2% soy PLs (soybean phospholipids group, SP). The SEM represented Scanning electron microscope.

Histopathological observations of H&E and Oil Red O staining of the livers were carried out to evaluate the protection of samples from HFD-induced liver damage (Figure 1). The liver tissue structure of rats in the control group was integrated. Hepatic cells were arranged regularly, the nucleus structure was clear, the hepatic lobular structure was normal, and hepatic cells had no pathological changes such as steatosis and swelling. As expected, the liver tissue photomicrographs of HFD rats in the model group showed lamellar and diffuse steatosis. The hepatic cells were obviously swollen. Nuclei were mostly squeezed to one side, and some showed a tendency to deform and disappear. The cytoplasm was filled with fatty vacuoles of different sizes. In the EP group, such pathological changes in the liver were not obvious. The arrangement of hepatocytes was relatively close compared with the other groups. Further, the liver lobules and cell structure were normal. In the SP group, a few hepatocytes showed slight steatosis and a few lipid droplets could be seen in the cytoplasm. The arrangement of hepatocytes was relatively close. The lobules of the liver were normal, and the structure of hepatocytes tended to be normal. The results of scanning electron microscopy were also very consistent with the previous results. The histopathological results indicate that the administration of PLs could distinctly counter hepatic steatosis caused by an HFD. Animal-derived egg yolk PL is more effective than plant-derived soybean in preventing liver cell degeneration and fat accumulation. These findings, together with the test results above, indicate that PLs can protect liver tissues from HFD-induced liver steatosis in rats.

### 3.4. Effects of Dietary PLs on Lipid Metabolism

Previous studies showed that an HFD affects lipid metabolism disorders [15]. The complex association between abdominal fat tissue and the liver is important for the development of NAFLD, but the mechanisms remain to be fully elucidated. Lipid profiles were assessed, and the results showed that TG in serum and TG and TC in the liver in the model group were significantly higher than that in the control group (Table 3). Hyperlipidemia already existed in these HFD-fed rats. The serum HDL-C level was significantly lower in the model group than that in the control group (*p* < 0.01). The serum TG levels in the EP and SP groups were significantly decreased compared with HFD-fed rats. The hepatic TC level in the EP group was significantly higher than that in the model group (*p* < 0.01), whereas the SP group followed the opposite trend (*p* < 0.01). The serum TG and LDL-C levels in the model group were slightly higher than that in the other three experimental groups, but no significant differences were noted (*p* > 0.05). These data indicate that PL intake could attenuate the formation of liver fat in HFD-fed rats by regulating the serum TG and HDL-C levels.

Compared with the HFD-fed rats, the choline and acetylcholine levels in the serum and brain from the control group rats were significantly increased. Moreover, an HFD with EPLs increased the serum choline and acetylcholine levels compared with the model group. Hepatic choline content in the SP and EP groups were significantly higher than that in the model group (*p* < 0.05). Acetylcholine is an important neurotransmitter in the central nervous system. The acetylcholine content in HFD-fed rats was significantly lower than that in the control group (*p* < 0.01). In addition, the acetylcholine content in the EP group was significantly increased compared with that in the model group (*p* < 0.05). The acetylcholine content in the SP group was also increased (*p* > 0.05). In contrast, we detected the PL content in serum. The consumption of EPLs effectively increased the PL content in serum (*p* < 0.05). The content of HDL-C, Cho, and ACH after EPL intervention in an HFD was significantly higher than that of SPLs (*p* < 0.05). All these results indicate that EPLs and SPLs counter choline and acetylcholine changes in the serum and liver.

### 3.5. Oxidation and the Anti-Inflammatory Factor

**Figure 2:**
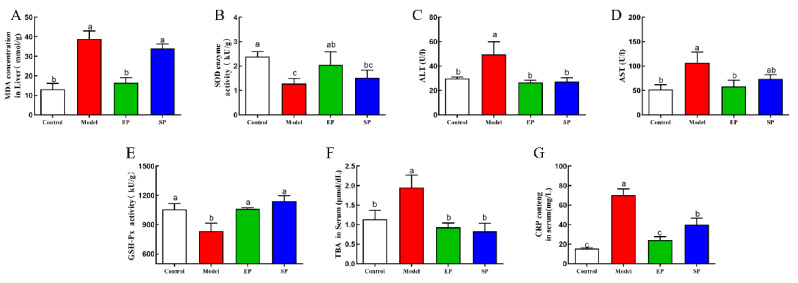
Effect of phospholipids on MDA, SOD, AST, ALT, NEFA, GSH-PX, TBA, and CRP. (**A**) MDA concentration in serum; (**B**) SOD enzyme activity in liver; (**C**) ALT enzyme activity in liver; (**D**) AST enzyme activity in serum; (**E**) GSH-Px enzyme activity in serum; (**F**) TBA concentration in serum; (**G**) CRP content in serum. An HFD containing 2% egg PLs (egg yolk phospholipids group, EP), and an HFD containing 2% soy PLs (soybean phospholipids group, SP). The different letters indicate significant difference at *p* < 0.05 determined by ANOVA (Duncan’s test).

Oxidative stress is one of the factors that causes hepatic dysfunction [16,17]. As shown in Figure 2, an HFD significantly promoted formation of MDA, reduced activities of enzymes including SOD and GSH-Px in the liver, and increased activities of enzymes ALT and AST in the serum. Notably, both EPL and SPL treatment significantly inhibited the increase of hepatic MDA level and attenuated the depletion of the antioxidant defense system induced by an HFD in comparison with the model group, in which EPLs exhibited a better effect than SPLs. In addition, the serum CRP and TBA levels in the EP and SP group rats were significantly decreased, and the CRP values were significantly lower than that in the SP group (*p* < 0.05). These results indicated that PLs inhibited inflammation and limited oxidative stress caused by an HFD.

### 3.6. Change in the Hepatic Fatty Acid Profile

**Figure 3:**
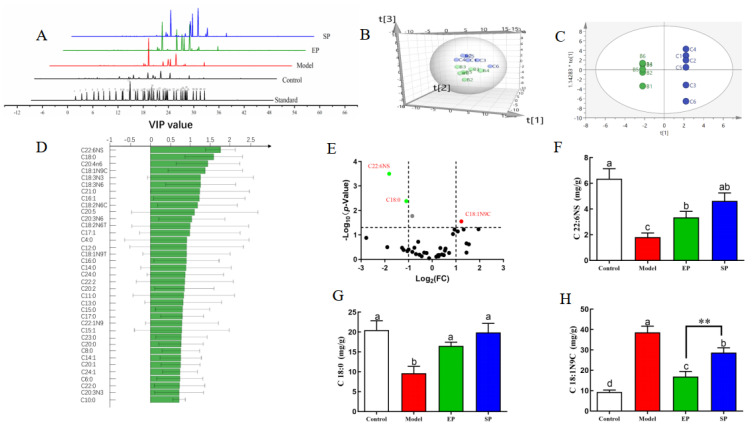
Treatment with phospholipid changes in hepatic fatty acid profiles. (**A**) Total ion chromatogram of liver samples and 37 fatty acid methyl esters standard, (**B**) PCA score 3D plot and (**C**) OPLS-DA score plot, (**D**) VIP scores of OPLS-DA, and (**E**) volcano plot for liver fatty acids of normal and HFD-fed rats. Potential biomarkers of fatty acid metabolism in HFD rats: (**F**) C22:6NS; (**G**) C18:0 of mouse liver; and (**H**) C18:1N9C of mouse liver. Peaks of the total ion chromatogram: (1) C4:0, (2) C6:0, (3) C8:0, (4) C10:0, (5) C11:0, (6) C12:0, (7) C13:0, (8) C14:0, (9) C14:1, (10) C15:0, (11) C15:1, (12) C16:0, (13) C16:1, (14) C17:0, (15) C17:1, (16) C18:0, (17) C18:1N9T, (18) C18:1N9C, (19) C19:0, (20) C18:2N6T, (21) C18:2N6C, (22) C20:0, (23) C18:3N6, (24) C20:1, (25) C21:0, (26) C20:2, (27) C22:0, (28) C20:3N6, (29) C22:1N9, (30) C20:3N3, (31) C20:4N6, (32) C23:0, (33) C22:2, (34) C24:0, (35) C20:5, (36) C24:1, (37) C22:6NS. An HFD containing 2% egg PLs (egg yolk phospholipids group, EP), and an HFD containing 2% soy PLs (soybean phospholipids group, SP). The different letters indicate significant difference at *p* < 0.05 determined by ANOVA (Duncan’s test). ** *p* < 0.01 significant EP to SP determined by Student’s *t*-test.

GC was used to determine the content and composition of liver fatty acids among the experimental groups. Figure 3A provides a chromatogram for each group. Multivariate pattern recognition analysis was used to directly visualize systematic variation in the fatty metabolome. The scores of PCA and OPLS-DA showed that the hepatic fatty acid composition of control rats was different from that of the model group, indicating that the HFD induced changes in liver fatty acids (Figure 3B,C). The variable importance in projection (VIP) score of fatty acids indicated that the liver fatty acids C22:6NS, C18:0, C20:4N6, C18:1N9C, C21:0, C18:3N3, and C18:3N6 were the top fatty acids, leading to group separation considered as the candidate pool for classification (Figure 3D). To accurately determine differential composition, candidate markers were also cross-selected by examining the volcano map considering a fold change threshold of 1.5 and a statistical P-value less than 0.05. Finally, docosahexaenoic acid (DHA, C22:6NS), octadecanoic acid (stearic acid, C18:0), and oleic acid methyl ester (methyl oleate, C18:1N9C) were identified as potential biomarkers for HFD-induced liver steatosis. Liver fatty acid C18:1N9C of SD rats fed with an HFD increased significantly (*p* < 0.01) (Figure 3F,G). However, after PL intervention, the C18:1N9C levels decreased in HFD-induced rats by 43.08% (*p* < 0.05) and 26.72% (*p* < 0.09). From another perspective, hepatic fatty acids C18:0 and C22:6NS in HFD rats significantly decreased (*p* < 0.001). Conversely, the contents of C18:0 and C22:6NS significantly increased in the EP and SP groups. Respectively, the EP and SP groups were lower than in the control group (*p* > 0.05).

## 4. Discussion

Defective lipid metabolism promotes hyperlipidemia and ectopic lipid accumulation [4]. It may be an underlying factor for coronary heart disease, myocardial infarction [18], atherosclerosis [19], and other diseases. Disorders of lipid metabolism cause lipid accumulation in non-adipose tissues such as liver, heart, kidney, muscle, and pancreas and produce lipotoxic effects on these organs or systems [19]. According to survey data, twelve million people die each year due to cardiovascular and cerebrovascular diseases globally [20]. The circulating lipid in the bloodstream directly and comprehensively reflects lipid metabolism in the body [21]. The main clinical manifestations of hyperlipidemia are abnormally elevated serum TC, TG, and LDL-C and abnormally reduced serum HDL-C. We selected PLs to investigate their impact on hepatic steatosis, metabolism of PLs with choline and acetylcholine, and fatty acid metabolism in rats fed an HFD with 2% of PLs. Our results confirmed that the administration of an HFD for four weeks induced accelerated weight gain, hepatic steatosis, and changes in liver fatty acid profiles, PL distribution, and PL metabolism, consistent with recent studies.

According to Oil red O staining, there was also an increase in neutral lipid accumulation in the liver. The liver fatty acid profile was studied via GC analysis. The differential fatty acid screen revealed that docosahexaenoic acid (DHA, C22:6NS), octadecanoic acid (stearic acid, C18:0), and methyl oleate (methyl oleate, C18:1N9C) could be used as biomarkers for PLs to regulate the disruption of lipid metabolism caused by an HFD. Han and co-workers suggested that increased levels of hepatic fatty acids contributed to triacylglycerol accumulation and lipotoxicity, which supported our results [7,22]. Our study suggested that the levels of hepatic C22:6NS and C18:0 in HFD-treated rats were significantly reduced, and C18:1 was significantly elevated as compared with the level in rats fed a normal diet. Both PL treatments were effective in balancing the fatty acid metabolic disorder caused by an HFD. The liver pathological sections showed that an HFD caused the accumulation of liver lipids, and the intervention of PLs had an improved effect.

Previous studies indicated that PLs can counter hyperlipidemia [23,24]. However, few studies compared PLs from different sources. The present study showed that EPLs suppressed trends of weight gain, whereas SPLs showed the opposite effect. Moreover, egg and soy PLs both showed a decreased liver and abdominal fat weight compared to the high-fructose diet fed group. However, the results in the EP group animals showed a greater effect than the SP group animals. The ingestion of excess fat exceeds the body’s ability to catabolize lipids, resulting in abdominal fat accumulation and liver hypertrophy [25]. The serum and hepatic TG levels decreased after consuming SPLs. The serum and hepatic TG levels were significantly decreased in the EP group rats. Our results illustrated that animal-derived egg yolk PLs reduced the atherogenic and coronary artery indices (*p* < 0.05), indicating that egg PLs may reduce the risks of cardiovascular and cerebrovascular diseases. However, we observe that the serum TC level was increased significantly in egg yolk PL treatment, which is slightly different from the previous experimental results [17]. HDL-C, synthesized mainly in the liver, is an anti-atherosclerotic lipoprotein that transports cholesterol from extrahepatic tissues to the liver [22]. We speculated that the phenomenon of transient high levels of cholesterol in the liver occurred due to the stimulation of HDL-C transport by high serum TC concentrations.

Numerous clinical data have shown that the development of hyperlipidemia is closely related to oxidative stress [23]. The occurrence of hyperlipidemia is accompanied by an increase in oxygen free radicals and peroxidative damage [26], which is consistent with the results of this paper. The antioxidant effect of PLs has been confirmed by numerous studies [27]. PLs and their oxides reduce mitochondrial damage and apoptosis [28]. In vivo, free radicals induce lipid peroxidation, and a final oxidation product is malonaldehyde [29]. Our study found that EPLs, compared with SPLs, exhibited antioxidation and anti-inflammatory activity that could decrease the hepatic MDA content and the serum TBA and CRP levels. Moreover, PLs, by preventing the negative effects of the high-fructose diet, ameliorate liver damage and decrease circulating transaminases. These results further suggest that exogenous PL can counteract lipid metabolism disorders and inflammation caused by HFD.

Several studies indicate that choline as a neurotransmitter precursor has significant effects in promoting neural signal transmission, improving brain vitality, delaying aging [14], and preventing vascular sclerosis [15,26], as well as antithrombotic effects [30]. Our results indicate a strong inverse relationship between the increase in PLs and changes in the fractions of choline and related compounds. An HFD may cause cognitive decline, and some studies have suggested that such a decline is associated with a decrease in choline content in brain tissue [31]. The results of this study further suggest that an HFD could cause disorders in the levels of PLs and metabolites in the brain. With the addition of PLs in the diet, the levels of choline and acetylcholine in blood, liver tissue, and brain tissue increased significantly.

## 5. Conclusions

The present study re-demonstrated that PL administration showed protective effects on HFD-caused liver steatosis in rats. In addition, PLs were associated with reduced serum oxidative stress, inflammation markers, and liver fatty acid profile disorder caused by an HFD. Compared with SPLs, EPLs have a certain therapeutic effect on hyperlipidemia and hepatic steatosis caused by the HFD that is prevalent today. PLs represent a potential nutritional strategy to treat hyperlipidemia.

## Figures and Tables

**Table 1:** Composition and mass percentage of experimental diets (%).

Components	Control Group	High-Fructose Group	EP	SP
Background diet	100	0	0	0
Sucrose	0	45.4	45.4	45.4
Casein	0	20	20	20
β-corn starch	0	15	15	15
Cellulose	0	5	5	5
Mineral mixture (AIN-76)	0	3.5	3.5	3.5
Vitamin blend	0	1	1	1
Corn oil	0	10	8	8
Soy bean phospholipid	0	0	0	2
Egg yolk phospholipid (PL-100M)	0	0	2.04	0
Cholesterol	0	0.1	0.06	0.1
SUM	100	100	100	100

Note: Commercial standard chow (control group), high-fructose group (model group), an HFD containing 2% egg PLs (egg yolk phospholipids group, EP), and an HFD containing 2% soy PLs (soybean phospholipids group, SP).

**Table 2:** Growth paraments and tissue weight in each group.

Control			High-Fructose Group	EP	SP
Growth parameters					
	Body weight, initial (g)	101.03 ± 8.31	99.57 ± 5.91	102.59 ± 7.94	99.79 ± 5.72
	Body weight, final (g)	257.70 ± 7.98 ^c^	281.65 ± 5.89 ^a^	274.56 ± 4.27 ^b^***	290.46 ± 6.51 ^a^
	Food intake (g/d)	16.52 ± 0.11	16.37 ± 0.12	16.44 ± 0.32	16.45 ± 0.25
	Food efficiency (%)	34.81 ± 0.88 ^c^	38.73 ± 1.17 ^ab^	37.05 ± 1.42 ^b^**	39.97 ± 2.25 ^a^
	Liver weight (g)	8.32 ± 0.81 ^b^	10.75 ± 1.39 ^a^	9.08 ± 0.54 ^b^	8.88 ± 0.53 ^b^
	Abdominal fat weight (g)	0.90 ± 0.40 ^c^	3.66 ± 0.78 ^a^	2.37 ± 0.58 ^b^*	3.55 ± 1.12 ^a^

Note: Data are presented as mean ± SEM (*n* = 6). An HFD containing 2% egg PLs (egg yolk phospholipids group, EP), and an HFD containing 2% soy PLs (soybean phospholipids group, SP). The different letters indicate significant difference at *p* < 0.05 determined by ANOVA (Duncan’s test). * *p* < 0.05. ** *p* < 0.01, *** *p* < 0.001 significant difference compared to SP group determined by Student’s *t*-test.

**Table 3:** Serum and hepatic lipids in each group.

		Control	High-Fructose Group	EP	SP
Serum parameters					
	TG (mg/dL)	27.40 ± 5.93 ^b^	39.07 ± 8.43 ^a^	24.73 ± 7.34 ^b^	30.07 ± 7.45 ^b^
	TC (mmol/L)	14.74 ± 4.80	15.85 ± 2.64	16.40 ± 3.15	17.08 ± 6.53
	HDL-C (μmol/dL)	32.38 ± 5.09 ^ab^	19.50 ± 4.28 ^c^	35.70 ± 11.62 ^a^*	25.37 ± 2.94 ^bc^
	LDL-C (μmol/dL)	8.32 ± 0.79 ^b^	11.20 ± 1.08 ^a^	8.79 ± 0.95 ^a^	8.43 ± 1.70 ^a^
	FFA (mmol/dL)	19.48 ± 9.72 ^c^	65.03 ± 10.87 ^a^	30.74 ± 13.77 ^bc^	39.96 ± 14.21 ^b^
	PLs (mg/dL)	110.93 ± 48.36 ^b^	128.1 ± 75.27 ^b^	189.77 ± 44.56 ^a^	140.6 ± 42.15 ^ab^
	Cho (nmol/mL)	13.59 ± 3.03 ^a^	6.44 ± 0.60 ^c^	10.53 ± 1.71 ^ab^*	9.42 ± 1.31 ^bc^
	ACH (nmol/mL)	2.52 ± 0.65 ^ab^	0.92 ± 0.46 ^c^	3.49 ± 0.58 ^a^**	1.81 ± 0.61 ^bc^
Hepatic parameters					
	TG (mg/g)	3.81 ± 2.16 ^c^	17.74 ± 3.01 ^a^	7.59 ± 4.89 ^bc^	9.65 ± 5.28 ^b^
	TC (μmol/g)	8.80 ± 3.37 ^c^	38.22 ± 3.95 ^b^	44.06 ± 4.53 ^a^	15.03 ± 6.58 ^ab^
	Cho (nmol/g)	24.9 ± 6.67 ^a^	13.24 ± 1.13 ^b^	18.86 ± 3.81 ^ab^	15.18 ± 7.16 ^b^
	ACH (nmol/g)	10.9 ± 3.76 ^a^	4.89 ± 1.17 ^b^	8.52 ± 0.13 ^ab^	6.64 ± 0.56 ^b^

Note: Data are presented as mean ± SEM (*n* = 6). An HFD containing 2% egg PLs (egg yolk phospholipids group, EP), and an HFD containing 2% soy PLs (soybean phospholipids group, SP). The different letters indicate significant difference at *p* < 0.05 determined by ANOVA (Duncan’s test). * *p* < 0.05, ** *p* < 0.01 significant difference compared to SP group determined by Student’s *t*-test.

## Data Availability

Not applicable.

## Supplementary Materials

The following are available online at https://www.mdpi.com/article/10.3390/foods10071569/s1, Table S1: Main fatty acid compositions of egg yolk and soybean phospholipids (%).

## Abbreviations

ALT	Alanine transaminase
ANOVA	Analysis of variance
AST	Aspartate transaminase
EPLs	Egg yolk phospholipids
FAME	Fatty acid methyl esters
FFA	Free fatty acid
FT-IR	Fourier transform infrared spectroscopy
GC	Gas chromatography
H&E	Hematoxylin and eosin
HFD	High-fructose diet
PCA	Principal component analysis
SEM	Scanning electron microscope
SD	Sprague Dawley
SPLs	Soya bean phospholipids
TC	Total cholesterol
TG	Triglyceride
VIP	Variable importance in projection
