# Supplementary material for: Comparison of Egg Yolk and Soybean Phospholipids on Hepatic Fatty Acid Profile and Liver Protection in Rats Fed a High-Fructose Diet
Source: Foods. 2021 Jul 6;10(7):1569. doi: 10.3390/foods10071569 (PMC8307941; doi:10.3390/foods10071569)
Supplement: Supplementary file 1 [file foods-10-01569-s001.zip › foods-1216139-supplementary.pdf]

Table S1 Main fatty acid compositions of egg yolk and soybean phospholipids (%)

	EPLs	SPLs
C16:0	29.87 ± 0.20**	15.46 ± 0.71
C16:1	1.04 ± 0.03**	0.09 ± 0.01
C18:0	15.44 ± 0.38**	4.43 ± 0.11
C18:1	26.43 ± 0.56**	15.93 ± 0.20
C18:2	15.14 ± 0.42	54.55 ± 0.06**
C20:0	N.D.	0.24 ± 0.01
C18:3	0.09 ± 0.03	6.36 ± 0.06**
C22:0	N.D.	0.46 ± 0.03
C20:3	0.42 ± 0.03	N.D.
C20:4	6.52 ± 0.35	N.D.

Note：N.D. means no detected; Data are presented as mean ± SEM (n=6). * $P<0.05$. ** $P<0.01$, significant difference compared to SPLs determined by Student’s t-test. EPLs represented the Egg yolk phospholipids and SPLs represented the soybean phospholipids.
